# Risk factors predicting the development of diabetes mellitus and metabolic syndrome following gestational diabetes mellitus

**DOI:** 10.3906/sag-2002-65

**Published:** 2021-04-30

**Authors:** Bülent CAN, Sema ÇİFTÇİ, Gülşah YENİDÜNYA YALIN, Nevin DİNÇÇAĞ

**Affiliations:** 1 Department of Internal Medicine, Division of Endocrinology and Metabolism, Faculty of Medicine, İstanbul Medeniyet University, İstanbul Turkey; 2 Department of Endocrinology and Metabolism, Bakırköy Sadi Konuk Training and Research Hospital, İstanbul Turkey; 3 Division of Endocrinology and Metabolism, Department of Internal Medicine, Faculty of Medicine, İstanbul University, İstanbul Turkey

**Keywords:** Diabetes, metabolic syndrome, gestational diabetes, insulin resistance

## Abstract

**Background/aim:**

To determine risk factors associated with the development of insulin resistance, type 2 diabetes mellitus (T2DM), and metabolic syndrome (MetS) in gestational diabetes mellitus (GDM) patients 10 years after giving birth.

**Materials and methods:**

Medical records of patients with former GDM were screened. Eligible patients were invited to the hospital to obtain information about their present health status. Patients with pregestational diabetes and patients with multiple pregnancies were excluded. A total of 67 women formed the study group. American Diabetes Association (ADA) and International Diabetes Federation (IDF) criteria were used to define T2DM and MetS, respectively.

**Results:**

A total of 27 patients developed diabetes (40.3%) and 35 patients (52%) developed MetS. T2DM developed, on average, 4.8 years after delivery. There was a significant difference between diabetic and nondiabetic patients in terms of insulin use during pregnancy (P < 0.001). Women who developed diabetes within 10 years after giving birth were observed to have significantly higher fasting plasma glucose on oral glucose tolerance test during their pregnancy (P = 0.007). Current and pregestational body mass indices had a significant effect on the development of MetS (P = 0.003 and P = 0.027, respectively).

**Conclusion:**

In this long-term study, we found that patients with high fasting plasma glucose (FPG) and insulin requirement during pregnancy are at an increased risk of developing T2DM, while pregestational obesity is predictive of progression to MetS. Identifying and targeting high-risk individuals may delay and possibly prevent T2DM and MetS.

## 1. Introduction 

Gestational diabetes mellitus (GDM) is characterized by glucose intolerance with first recognition during pregnancy. The global prevalence of GDM ranges from 5% to 20% depending on the study population [1]. Women with GDM may develop type 2 diabetes mellitus (T2DM), prediabetes, metabolic syndrome (MetS), and cardiovascular disease in the years following their pregnancy [2–4]. 

Pregnancy is associated with a physiological insulin resistance, particulary in the second trimester, due to placental hormones such as human placental lactogen, progesterone, cortisol, growth hormone, and prolactin. GDM patients are shown to have insulin resistance combined with impaired secretion of insulin due to a defect in pancreatic β-cell function. Thus, the stress of pregnancy may trigger clinical diabetes in a predisposed individual [5].

In addition to being a major cause of kidney failure, coronary artery disease, and stroke, T2DM is among the first seven causes of disease-related deaths worldwide [6]. As well as being a constellation of cardiovascular risk factors, MetS is also associated with increased morbidity and mortality [7]. Thus, prevention of both conditions is of utmost importance worldwide. Early recognition of high-risk patients in the preclinical period, and appropriate preventive strategies may reduce the risk of progression to T2DM and MetS. Therefore, the purpose of this study was to determine risk factors associated with the development of insulin resistance, T2DM, and MetS in GDM patients 10 years after giving birth.

## 2. Subjects and methods

### 2.1. Study population and design

The study was undertaken in Istanbul Faculty of Medicine, department of Endocrinology and Metabolism. The inclusion criteria were as follows: 1) women who were diagnosed with GDM 10 ± 2 years previously and 2) women who were at least 18 years old at the time of pregnancy. Medical records of eligible patients were screened retrospectively. A total of 260 patients were screened. Patients with pregestational diabetes and patients with multiple pregnancies were excluded. Patients who met the eligibility criteria were called and invited to the hospital. A total of 67 women, who fulfilled the eligibility criteria and gave written informed consent, formed the study group. A flowchart of participation is shown in Figure. 

**Figure 1:**
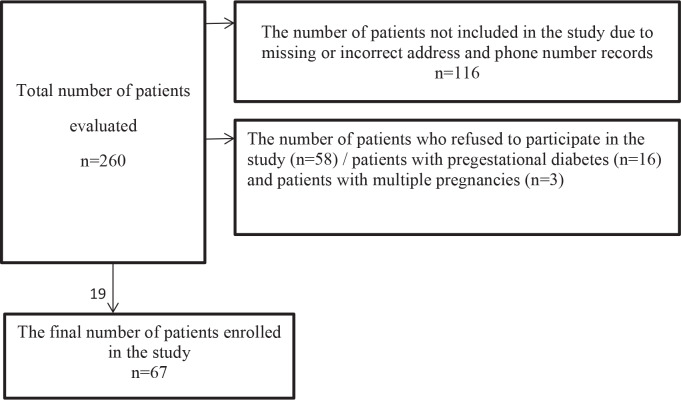
Flowchart of participation.

The institutional review board approved the study protocol (protocol no: 12-510). The study was conducted in compliance with the Declaration of Helsinki. 

All patients underwent physical examination including systolic and diastolic blood pressure. The weight, height, body mass index (BMI), waist circumference, and hip circumference of all patients were recorded. Blood pressures were measured twice in a sitting position after at least 10 min of rest with a mercury sphygmomanometer. BMI was calculated as weight (kg) divided by height in meters squared (m²). Waist circumference was measured at the midpoint between the top of the iliac crest and the lower margin of the least palpable rib, and hip circumference was measured around the widest portion of the buttocks using a flexible tape [8]. 

GDM was defined as glucose intolerance with first recognition during pregnancy. Impaired fasting glucose (IFG) was defined as fasting plasma glucose (FPG) between 100 and 125 mg/dL (5.6 and 6.9 mmol/L), and impaired glucose tolerance (IGT) was defined as 2 h glucose between 140 and 199 mg/dL (7.8 and 11.0 mmol/L) during 75 g oral glucose tolerance test (OGTT). Patients were diagnosed with T2DM if they had one of the following: FPG ≥126 mg/dL (7.0 mmol/L) or 2 h glucose ≥200 mg/dL (11.1 mmol/L) during OGTT, or HbA1c ≥6.5% (48 mmol/mol) or a random plasma glucose ≥200 mg/dL (11.1 mmol/L) in a patient with symptoms of hyperglycemia [9].

MetS was defined as ethnicity-specific waist circumference plus any two of the following: High triglycerides >150 mg/dL (1.7 mmol/L), low HDL-cholesterol <50 mg/dL (1.29 mmol/L), receiving treatment for a lipid abnormality, systolic blood pressure ≥130 mmHg, diastolic blood pressure ≥85 mmHg, receiving treatment for hypertension, FPG ≥100 mg/dL (5.6 mmol/L), or previously diagnosed T2DM [10]. Insulin resistance (IR) was calculated using the following formula: HOMA-IR (Homeostasis model assessment of insulin resistance) = [Fasting plasma insulin (μU/mL) × FPG (mg/dL)] / 405 with a cut-off value of 2.6 [11]. 

Blood samples were drawn after an 8 h fast. Plasma glucose concentration was assessed using the hexokinase method with Abbott Architect ci16200 automatic analyzer (Diamond Diagnostics, Holliston, MA, USA). HbA1c was measured by a turbidimetric inhibition immunoassay (TINIA) (Roche Diagnostics, Mannheim,Germany). OGTT was performed in all patients who were not already diagnosed with diabetes. Patients were told to ingest at least 150 g/day of carbohydrates for 3 days prior to the test. OGTT was performed in the morning between 7 and 9 AM after 8 h overnight fasting. Blood samples were drawn before and 60, 120, and 180 min after the ingestion of 75 g glucose. 

### 2.2. Statistical analysis

After data distributions were tested, parametric distributions were expressed as mean ± standard deviation, nonparametric distributions were expressed as median (interquartile range), and categorical parameters were expressed as percentage. Chi-square tests were performed to compare categorical parameters. The independent samples t-test and Mann–Whitney U Test were used to compare noncategorical parameters between diabetic and normal glucose tolerance patients, and between MetS positive and negative patients. Pearson and Spearman correlations were used to determine the relationships between variables. Binary regression analysis was performed to determine the risk factors for MetS development. Statistical significance was set at P < 0.05. All statistical analyses were performed using the SPSS 21.0 version (Statistical Package for Social Sciences, IBM Corp., Armonk, NY, USA). 

## 3. Results 

Demographic characteristics and laboratory values of study participants are presented in Table 1. A total of 67 patients with previous GDM were analyzed 10 ± 2 years postpartum. A total of 27 patients developed diabetes (40.3%), 13 developed prediabetes (IFG and/or IGT) (19.4%), and 27 had normal glucose tolerance. T2DM developed, on average, 4.8 years after delivery. MetS developed in 52.2% (n = 35) of the patients. 

**Table 1:** Demographic characteristics and laboratory measurements of the participants.

	Mean ± SDor median (IQR)	Min-max
Age (years)· Current· At pregnancy	42.1 ± 5.331.8 ± 5.3	32.0–54.020.0–42.5
BMI (kg/m2)· Current · Before pregnancy	30.4 ± 5.326.7 ± 5.0	20.5–46.118.3–40.1
Weight gain during pregnancy (kg)	12.0 (70–15.0)	(–10.0)–27.0
Birth weight (kg)	3.45 (2.85–3.75)	1.50–5.50
Waist circumference (cm)	96.9 ± 11.5	70.0–125
Waist/hip ratio	0.88 ± 0.06	0.76–1.05
Smoking intensity (pack-years)	5.5 (4.25–20)	1–30
Blood pressure (mmHg)· Systolic· Diastolic	122 ± 1878 ± 10	90–20060–100
Total cholesterol (mg/dL)	198 ± 37	115–301
Triglyceride (mg/dL)	120 (79–148)	40–402
LDL-cholesterol (mg/dL)	121 ± 32	69–221
HDL-cholesterol (mg/dL)	49 (43–64)	30–88
HbA1c (%)	6.5 ± 1.5	5.3–13.8
C-peptide (ng/mL)	2.06 (1.1–3.1)	0.03–9.1
FPG during pregnancy (mg/dL)	95 (84–125)	53–243
HOMA-IR	1.69 (1.14–3.45)	0.40– 9.84
TSH (mIU/L)	1.95 (1.41–2.82)	0.42–7.6
fT4 (pmol/L)	14.7 ± 2.1	8.6–20.1

The results were calculated using logarithmic transformations. Mean ± SD; mean ± standard deviation.

Eleven women (16.4%) gave birth to a macrosomic baby, 44 (65.7%) underwent caesarean section, 13 (19.4%) had obstetric problems during pregnancy or labor, 27 (40.3%) had preterm labor, and 11 (16.4%) had babies with health issues (prolonged jaundice, hypoglycemia, asphyxia, and/or growth retardation). Fifty-four patients (80.6%) had a family history of DM. Thirty patients (44.8%) were treated with insulin during pregnancy. As for the patients’ current medication: 14 patients (20.9%) were on insulin treatment, 35 patients (52.2%) used an oral antidiabetic, 11 (16.4%) used an antihypertensive, and 5 (7.5%) used a lipid lowering drug.

There was no significant difference in the mean levels of BMI and HOMA-IR between diabetes-developing patients and nondiabetic patients. The rate of antihypertensive drug use was 29.6% in patients with diabetes and 7.5% in patients without diabetes (P = 0.022). As for obstetric histories, there was no significant difference between diabetic and nondiabetic patients in terms of weight gain during pregnancy, history of diabetes in a first degree relative, or fetal macrosomia (P > 0.05). However, there was a significant difference between diabetic and nondiabetic patients in terms of insulin use during pregnancy (P < 0.001) (Table 2). Of those patients with current DM, 77.8% used insulin during their pregnancy (n = 21/27). Excluding the 13 patients who had IFG and/or IGT, only 7 (25.9%) women with normal glucose tolerance used insulin while pregnant. Women who developed diabetes within 10 years after delivery were observed to have significantly higher FPG levels on OGTT during their pregnancy (Table 2). 

**Table 2:** Demographic characteristics, laboratory measurements, and obstetric history of patients with and without T2DM.

	Diabetic (n = 27)Mean ± SDor median (IQR)	Normal glucose tolerance(n = 27) Mean ± SDor median (IQR)	P value
Age (years)· Current· At pregnancy	43 ± 5.7 32 ± 5.7	41 ± 4.832 ± 5.0	ns†ns†
BMI (kg/m2)· Current· Before pregnancy	30.7 ± 5.626.5 ± 5.6	30.0 ± 5.726.4 ± 5.2	ns†ns†
Waist circumference (cm)	97.7 ± 11.1	95.1 ± 11.3	ns†
Blood pressure (mmHg)· Systolic· Diastolic	126 ± 2178 ± 11	118 ± 1676 ± 9	ns†ns†
Triglyceride (mg/dL)	125 (98–170)	105 (69–144)	ns††
HDL cholesterol (mg/dL)	49 (37–65)	54 (46–64)	ns††
HOMA-IR	1.9 (0.9–4.7)	1.5 (1.3–2.2)	ns††
C-peptide (ng/mL)	2.0 (0.7–3.2)	2.2 (1.5–3.3)	ns††
Birth weight of the infant (g)	3500 (2750–3750)	3450 (3150–3800)	ns††
FPG during pregnancy (mg/dL)	125 (96–152)	88 (81–100)	P = 0.007††
Weight gain during pregnancy	10 (7–15)	13 (9–17)	ns††
Smoking intensity (pack-years)	15 (5–21)	4.5 (1.75–10.25)	ns††
Family history of T2DM (%)	85.2	81.5	ns**
Insulin requirement during pregnancy (%)	77.8	25.9	P < 0.001**

The results were calculated using logarithmic transformations. Mean ± SD; mean ± standard deviation.Unless otherwise specified in the table, the variables show measurements at the time of the study. IQR: interquartile range; T2DM: type 2 diabetes mellitus. ns: nonsignificant. †

Subgroup analysis regarding the development of MetS revealed that weight gain during pregnancy and maternal age showed no significant difference (P = 0.051). On the other hand, current and pregestational BMI values showed a significant difference between MetS positive and MetS negative patients (Table 3). Binary regression analysis regarding obstetric risk factors revealed that fetal macrosomia, type of birth, time of birth, history of diabetes in a first degree relative, and insulin use in pregnancy had no significant effect on the development of MetS 10 years after delivery (Odds ratio; 95% CI and P values are: 0.483; 0.123–1.895, P = 0.297, 1.193; 0.450–3.162, P = 0.723, 1.202; 0.423–3.416, P = 0.730, 1.786; 0.504–6.335, P = 0.369, and 1.723; 0.592–5.018, P = 0.318, respectively).

**Table 3:** Demographic characteristics, laboratory measurements, and obstetric history of patients with and without MetS.

	MetS (+) (n = 35)Mean ± SD ormedian (IQR)	MetS (-) (n = 32)Mean ± SD ormedian (IQR)	P value
Age (year)· Current· At pregnancy	43 ± 4.733 ± 4.7	41 ± 5.631 ± 5.7	0.054†0.051†
BMI (kg/m2)· Current· Before pregnancy	32.8 ± 5.028.2 ± 5.1	27.8 ± 4.424.7 ± 4.2	0.003†0.027†
Weight gain during pregnancy (kg)	10.0 (6.0–14.0)	14.0 (9.2–15.7)	0.051††
Waist/hip ratio	0.91 ± 0.05	0.85 ± 0.05	0.006†
Smoking intensity (pack-years)	5.0 (2.0–20.0)	6.0 (4.5–20.0)	0.749††
HbA1c (%)	7.1 ± 1.9	5.9 ± 0.7	<0.001†
HOMA-IR	2.75 (1.94–4.75)	1.24 (0.73–1.64)	<0.001††
C-peptide (ng/mL)	2.77 (2.06–3.60)	0.70 (0.34–1.31)	<0.001††
Birth weight of the infant (g)	3350 (2850–3650)	3450 (3040–3937)	0.580††
Family history of DM (%)	86	75	0.268**
Insulin requirement during pregnancy (%)	51	37	0.252**

The results were calculated using logarithmic transformations. Mean ± SD; mean ± standard deviation. P < 0.05 statistically significant. Significant P values are shown in bold. Unless otherwise specified in the table, the variables on the table show measurements at the time of the study. MetS: metabolic syndrome †The independent samples t-test was used, ††

Obstetric history and BMI of patients with and without insulin resistance are shown in Table 4. 

**Table 4:** Comparison of BMI and obstetric history of patients with and without insulin resistance.

	HOMA-IR		P value
	≥2.6 Mean ± SD(n = 24)	<2.6 Mean ± SD(n = 42)	
BMI (kg/m2)	32.8 ± 4.9	28.9 ± 5.1	0.003*
Pregestational BMI	28.7 ± 4.9	25.2 ± 4.6	0.002*
Age at pregnancy	33.5 ± 4.1	30.8 ± 5.7	0.003*

Mean ± SD; mean ± standard deviation. P < 0.05 statistically significant. Significant P values are shown in bold.

## 4. Discussion 

In this study, we found that approximately 60% of prior GDM patients developed diabetes or prediabetes while 50% developed MetS over a period of 10 years. Our finding is in line with literature where the cumulative incidence of T2DM development over 5 years is reported to be approximately 50% [12,13]. Our rate of progression to MetS is also consistent with previous reports [14,15]. Current guidelines recommend screening GDM patients with OGTT 4–12 weeks after delivery and then every 1–3 years [16]. However, there is no consensus as to how long GDM patients should be monitored. In our study, diabetes developed, on average, 4.8 years after delivery. In agreement with this result, the rate of GDM progression to T2DM is reported to be highest during the first 5 years after delivery, with a slower increase after 10 years [12]. We therefore recommend annual screening for the first 5 years after GDM for high-risk patients. 

Fasting plasma glucose on OGTT is the factor most commonly linked with progression to T2DM [12]. Furthermore, BMI, waist circumference, gestational insulin use, and early gestational age at the time of GDM diagnosis have all been associated with the development of T2DM in patients with GDM [17,18]. A retrospective cohort study reported that maternal age at delivery and birth weight of the baby were also associated with diabetes development [19], but contradictory findings exist [20]. In line with the literature, our data suggest that progression to T2DM is mainly determined by higher FPG levels and insulin use during pregnancy. Of the patients with current T2DM, 77.8% were prescribed insulin during pregnancy. This ratio was 25.9% for those with normal glucose tolerance. Women with GDM have been shown to have chronic insulin resistance and β-cell dysfunction [21]. Elevated FPG during pregnancy suggests insulin resistance, while insulin requirement indicates an impaired β-cell function. As a result, FPG and insulin use may be related to the severity of GDM and hence predict the likelihood of progression to T2DM. There is evidence that T2DM and the resulting cardiovascular disease can be prevented with lifestyle changes or medical therapy [13,22]. Also of note is that awareness improves adherence to lifestyle changes [23]. We therefore recommend that high-risk patients (i.e. patients with higher FPG and those who require insulin treatment during pregnancy) be informed about their individual risk of developing diabetes.

In our study, BMI, HOMA-IR scores, weight gain during pregnancy, history of diabetes in a first degree relative, and fetal macrosomia were not related to the development of T2DM. Similarly, Rayanagoudar et al. report in their systematic review that gestational glycemic status is the main determinant of T2DM risk in the future, and that gestational weight gain or macrosomic infant do not increase the risk [24]. Also in line with our findings, most studies have failed to establish a relation between family history of T2DM and progression to diabetes [12]. However, several studies have linked obesity to the future risk of T2DM [25]. GDM patients who come to our hospital are monitored closely and are asked to adhere to a strict diet, which may have limited their weight gain during, and after pregnancy. Ethnicity, dietary habits, and the prevalence of obesity in our country may also have affected our results.

We found that patients with higher HOMA-IR scores had significantly higher BMI, pregestational BMI, and maternal age compared to patients without IR. IR is a well-known precursor of T2DM. However, our results suggest that IR alone has limited power to predict transition from GDM to T2DM. This is probably due to other factors involved in the transition, such as pancreatic β-cell reserve and the polygenic inheritance of T2DM [9].

As for the relation between IR and dyslipidemia, we found that patients with HDL <50 mg/dL and triglyceride ≥150 mg/dL had significantly higher HOMA-IR scores than patients with HDL ≥50 mg/dL and triglyceride <150 mg/dL. Presence of dyslipidemia, antihypertensive drug use, or smoking status did not differ among these two groups. Our results were as expected, since the typical dyslipidemia of insulin resistant state involves hypertriglyceridemia and low HDL. 

We found that diabetic patients had a higher rate of antihypertensive drug use. Diabetes and hypertension were possibly found together in the same individual due to shared etiological factors including obesity, inflammation, and oxidative stress. Moreover, insulin resistance is known to be effective in the development of both T2DM and hypertension. Coexistence of T2DM and hypertension can also be explained genetically, since there is evidence indicating that variants of angiotensinogen and adrenomedullin gene are associated with both conditions [26]. 

Metabolic syndrome is a cluster of abdominal obesity, insulin resistance, dyslipidemia, and hypertension. Previous studies have shown that the development of MetS in GDM patients is associated with current and pregestational BMI [27,28]. In agreement with literature, current and pregestational BMI had a significant effect on the development of MetS in our study. Adipose tissue is known to secrete adipokines, which are involved in inflammatory processes. It is probably due to these adipokines that subclinical inflammation, IR, and endothelial dysfunction, all of which lead to the development of MetS, are greater in previously obese GDM patients. There is also evidence suggesting that GDM and MetS may have a common genetic background. A relationship between GDM and the risk gene variants is similarly present in MetS [29]. Our results suggest that this genetic predisposition may be more evident in obese individuals. Since obesity is a modifiable risk factor, high-risk patients may benefit from lifestyle changes and medical intervention to prevent MetS.

A byproduct of insulin synthesis, C-peptide has previously been studied as a sensitive indicator of MetS [30]. Similarly, we found that C-peptide levels of MetS patients were significantly different compared to the group of patients without MetS. This difference may be explained by the fact that plasma C-peptide concentrations correlate better with β-cell function during insulin resistance. In addition, C-peptide is known to regulate inflammatory cytokines and may thus have a correlation with MetS, which, as previously mentioned, is a chronic low-grade inflammatory state [31]. The clinical implication of this easy laboratory tool is that it may be used to identify patients at risk of developing MetS. As we only had information on patients’ current C-peptide levels, further studies comparing C-peptide concentrations before and after pregnancy are needed to be able to draw conclusions.

In our study, obstetric risk factors such as fetal macrosomia, type of birth, time of birth, history of diabetes in a first degree relative, insulin use during pregnancy, weight gain during pregnancy, and maternal age had no significant effect on the development of MetS, which is in agreement with the literature [32]. The relationship between insulin use and T2DM development was probably not strong enough to be effective in the development of MetS, which is a *cluster* of several risk factors. 

Finally, our study has several limitations worth mentioning. First of all, the relatively small sample size was a limitation of this study. A larger study population could reveal novel associations that our study failed to demonstrate. Presence of cardiovascular risk factors following pregnancy was not evaluated due to the retrospective design of the study. Another limitation was lack of information about the glycemic control of GDM patients during pregnancy. However, our medical center is a university hospital with a highly experienced team of endocrinologists who check GDM patients on a weekly basis to ensure best possible glycemic control. It is also worth mentioning that the exclusion of pregestational diabetes was based on HbA1c values for only 18 GDM patients. For the rest of the patients, the exclusion was based on patient history. However, when we reevaluated GDM patients with high FPG, we found that only one of them developed post gestational T2DM, making pregestational diabetes an unlikely diagnosis. The only patient with high FPG who developed T2DM after pregnancy had a pregestational HbA1c of 5%; hence none of the patients were suspected to have pregestational diabetes.

Lack of objective data regarding the prevalence of MetS before pregnancy presents a major limitation to the study. Other than pregestational BMI and FPG values, MetS diagnosis was excluded based on patient history. Patients with established dyslipidemia, hypertension, diabetes, or with related drug use did not fulfill the eligibility criteria. Therefore, although not definite, patients included in the study were assumed to not have had MetS before pregnancy.

To the best of our knowledge, this is the first long-term study to associate GDM with both T2DM and MetS in our country. Previous GDM studies in literature mostly focus on the metabolic state at the early postpartum period or just a few years after delivery [33,34] while few studies present a long-term evaluation [19,20]. Our 10 year follow-up time was a strength of the study. 

In conclusion, effective postpartum follow-up of patients diagnosed with GDM is essential since GDM may progress to T2DM and MetS, both of which are major public health problems. We found in this long-term study that patients with high FPG and insulin requirement during pregnancy are at an increased risk of developing T2DM, while pregestational obesity is predictive of progression to MetS. Identifying and targeting high-risk individuals may delay and possibly prevent T2DM and MetS. Future prospective studies with larger study populations are warranted to clarify the contradictory findings in literature.
